# Accessing and sharing health information for post-discharge stroke care through a national health information exchange platform - a case study

**DOI:** 10.1186/s12911-019-0816-x

**Published:** 2019-05-03

**Authors:** Nadia Davoody, Sabine Koch, Ingvar Krakau, Maria Hägglund

**Affiliations:** 1 Department of Learning, Informatics, Management and Ethics, Health Informatics Centre, Karolinska Institutet, Tomtebodavägen 18 A, 171 77 Stockholm, Sweden; 2 Department of Medicine, Karolinska Institutet, Solnavägen 1, 171 77 Stockholm, Sweden; 3 Department of Womens and Childrens Health, Uppsala Universitet, Akademiska sjukhuset, 751 85 Uppsala, Sweden

**Keywords:** Health information exchange, eHealth services, Interoperability

## Abstract

**Background:**

Patients and citizens need access to their health information to get a retrospective as well as a prospective view on their care and rehabilitation processes. However, patients’ health information is stored in several health information systems and interoperability problems often hamper accessibility. In Sweden a national health information exchange (HIE) platform has been developed that enables information exchange between different health information systems. The aim of this study is to explore the opportunities and limitations of accessing and interacting with important health information through the Swedish national HIE platform.

**Methods:**

A single case study approach was used for this study as an in-depth understanding of the subject was needed. A fictive patient case with a pseudo-name was created based on an interview with a stroke coordinator in Stockholm County. Information access through the national health information exchange platform and available service contracts and application programming interfaces were studied using different scenarios.

**Results:**

Based on the scenarios created in this study, patients would be able to access some health related information from their electronic health records using the national health information exchange platform. However, there is necessary information which is not retrievable as it is either stored in electronic health records and eHealth services which are not connected to the national health information exchange platform or there is no service contract developed for these types of information. In addition, patients are not able to share information with healthcare professionals.

**Conclusion:**

The national Swedish HIE platform provides the building blocks needed to allow patients online access to their health information in a fragmented and distributed health system. However, more complex interaction scenarios allowing patients to communicate with their health care providers or to update their health related information are not yet supported. Therefore it is of great importance to involve patients throughout the design and evaluation of eHealth services on both national and local levels to ensure that their needs for interoperability and information exchange are met.

**Electronic supplementary material:**

The online version of this article (10.1186/s12911-019-0816-x) contains supplementary material, which is available to authorized users.

## Background

Information and communication technology (ICT) has the potential to improve efficiency, satisfaction, and quality of care [1]. Currently, healthcare is shifting from organisation-centred to patient-centred or citizen-centred care and ICT can play an essential role in this paradigm shift as it can empower patients and citizens by e.g. giving them access to their health information [2]. In addition, patient participation has become more common in healthcare and patients and citizens request access to their health data to enable their involvement in decision making, engagement with their healthcare information and control of their care processes [3, 4]. With the aim to increase the engagement of patients in promoting health and managing illness one prominent strategy taken by many care providers and policymakers, especially in the US, is to give patients access to parts of their medical documentation, e.g. laboratory results and medication lists through patient portals. More recently, the trend toward transparency accelerates as initiatives to provide patients with online access also to their full electronic health record (EHR), including clinician’s notes, spread both in the US with the OpenNotes project [5], and in Europe with e.g. the SUSTAINS project [6].

Several studies have explored patients’ and citizens’ online access to their health information and medical records [7–9]. These studies do not confirm care professionals’ concerns about negative effects such as patient worries that might be assumed following from patients accessing their health information online. Rather the studies indicate that having online access to health information will give patients the opportunity to check their past activities; be prepared for future actions, and develop new expectations. In addition, the results show that providing patients online access to their EHR offer increased satisfaction and improved patient safety [4].

However, there are some challenges in increasing transparency for patients especially for those with chronic or long-term conditions that require support from many different specialities and care providers. To provide patients with a holistic view of their health information, different health information system (HISs), such as different EHR applications, and eHealth services need to be able to communicate with each other and exchange information. Interoperability between different HISs is one of the main areas in medical informatics research and has long been in focus. The technology of the Good European Health Record (GEHR) [10] and Synapses/Synex [11, 12] projects (1992–1999) can be seen as a first mature approach to reach interoperability and integration of electronic health records (EHRs). Currently, there are several standards well under development. Health Level 7 (HL7) version 3 [13], including the HL7 Reference Information Model (RIM) and the Clinical Document Architecture (CDA) aim to structure and mark-up the clinical content for the purpose of exchange [14]. The European Standard for Electronic Health Record Communication EN13606, which has also become an international standard (ISO/EN 13606) [15] and openEHR [16] however, aim primarily to enable structured data capture without double entry and have information transfer as their secondary aim.

Based on the standards described above, there are several ongoing international initiatives to facilitate the implementation in practice. Integrating the Healthcare Enterprise (IHE) [17] is one example which provides specifications, tools and services for interoperability based on standards such as DICOM and HL7. As a response to the exploding market of personal health applications and mobile apps new approaches for connecting the HIS used within healthcare with personal health records are emerging. SMART (Substitutable Medical Applications and Reusable Technologies) on FHIR (Fast Health Interoperability Resources) [18] is an example of a platform enabling innovators to create apps and focusing on integration between medical apps into diverse electronic health record systems. A challenge in this area is the lack of regulations related to personal health applications which can create privacy issues [19].

In practice, there are existing experiences of standards implementation that have succeeded to provide access to clinical and health related information within a region e.g. Lombardy region in Italy [20] and openEHR implementation in Chinese hospitals [21, 22]. Yet, interoperability of HIS remains a problem in most healthcare systems and was identified as still being a major issue for usability of eHealth in Sweden in a study from 2013 [23].

### The Swedish national health information exchange platform

Sweden has chosen to implement a national health information exchange (HIE) platform [24] to facilitate the communication between different HISs and eHealth services. The national health information exchange platform enables a single point of connectivity for client applications, making all Swedish EHRs appear as a national, virtual EHR. Client applications may be targeted for patients, professionals, researchers, payers, byers and follow-up. The national HIE platform allows exchange of health care data between different HISs, care organisations, governmental agencies, patient communities and patients according to nationally defined *service contracts*. Some of the service contracts are adoptions of specifications for IHE profiles and Continua Guidelines and HL7 CDA is used as clinical model, through the HL7 Green CDA methodology [25]. Rather than having direct integration between HISs, all integration is with the national HIE platform which then redirects requests for information and transactions to the appropriate system.

In summary, the national HIE platform forwards the request message from a system or a service to the appropriate source systems, often e.g. EHR systems used by different care providers and returns the response, which may be aggregated from multiple sources [26].

In our previous studies a care and rehabilitation planning tool called ‘My Care Plan’ for post-discharge stroke patients has been developed [27–29] which is used in this study to explore the opportunities and limitations of accessing and interacting with important health information through the Swedish national HIE platform. This will provide important insights into strengths and weaknesses in the Swedish approach, or similar platforms, and requirements for further development in the future.

#### The health innovation platform

In parallel with the development and implementation of the national HIE platform, a Health Innovation Platform (HIP) [24] was developed within the research project “My care pathways” to facilitate open innovation and development of eHealth services for both caregivers and patients/citizens [30]. HIP is an application innovation portal providing instructions and code for accessing data and transactions according to the specifications in the national HIE platform. The idea is that developers, designers and entrepreneurs have access to the tools for development of eHealth services for healthcare and patients/citizens [24].

#### Patients’ online access to their EHR

As an example, one of the main service consumers currently in use is the national eHealth service “Journalen” giving patients’ access to their EHRs [31–33]. Starting off as a service connected to a specific EHR system in 2012, “Journalen” has since been migrated to connect to the national HIE platform and is now accessible to everyone in Sweden who has an account in the virtual national patient portal called 1177 Vårdguiden (1177 Healthcare guide’s e-services) [34]. In May 2018 more than 5 million people had set up accounts, which correspond to 50% of Sweden’s inhabitants. All these users therefore have access to the service “Journalen”, but whether or not they can actually access their health data depends on whether their care provider releases all information to the patient through their connection to the national HIE platform as a service producer [32, 35]. As of April 2018, all Swedish county councils and regions are connected as service providers, but they still provide different amounts of information [36, 37]. In total, almost 2 million persons had used “Journalen” to access their EHR by May 2018, and over 1 million log-ins are made each month [38].

Giving patients’ access to their EHR will provide them with information about past visits, laboratory results etc. Yet, the medical record notes provide mainly a retrospective perspective on care provided. For many patients with chronic or long-term conditions, a more prospective view is also required and can be provided through care plans [27]. Having both a retrospective and prospective view of health information is crucial for giving patients an overview of their journey throughout their care and rehabilitation processes.

### Care and rehabilitation planning

There are several definitions of what a *care plan* is. The national board of health and welfare in Sweden defines a care plan as “a health and social care plan that outlines health care for an individual patient” [39]. According to the Swedish national health handbook “care plan includes the diagnosis (problems, risks) and goals that have been determined with and for a patient, followed by actions/activities and prescriptions” [40].

Descriptions of digital or computerized care plans have however been mostly limited to two types; (1) tools for discharge planning when patients are transferred from in-hospital to primary care [41, 42], and (2) standard care plans for short term specialist care or chronic disease management [43, 44]. However, *individualized care plans* cover all aspects of a patient’s care and all care providers involved are increasingly used to ensure continuity and coordination of care, and initiatives to create tools for such care plans are increasing [45–47].

Rehabilitation plans are a type of individualized care plans used within rehabilitation to set goals and plan activities to achieve these goals. As the success of rehabilitation is very much dependent on the engagement and actions of the patient, rehabilitation planning has been an essential tool for increasing patients’ motivation and efforts [48, 49].

To provide patients with an overview of their care and rehabilitation processes, in our previous studies [27–29] an electronic care and rehabilitation planning tool for post-discharge stroke care was designed, “My Care Plan”.

#### The web-based tool ‘My Care Plan’

In Stockholm County Council, post-discharge stroke patients receive care from different care professionals including physicians, district nurses, nurses and a neurology team. The team include a physiotherapist, an occupational therapist, a counsellor, and a speech therapist. The home visits from the team can last up to one year. A rehabilitation plan is established together with the patient and potential next-of- kin at the patient’s home and is documented in the patient’s health record [50, 51]. The rehabilitation plan includes identifying the patient’s problems and defining the goals and activities. Currently, the rehabilitation plan for post-discharge stroke patients in Stockholm County is paper-based. In our previous study interaction points between post-discharge stroke patients and the care professionals throughout the care and rehabilitation processes were explored [52]. The information needs of patients were then identified and several potential eHealth services were suggested [27]. Subsequently, a care and rehabilitation planning tool was designed. The aim of the tool is to improve patient self-management and collaboration between different care professionals with patients and their next-of-kin by giving them access to necessary information and providing an overview of the care and rehabilitation plan.

The web-based tool provides a rehabilitation plan that includes *problems*, *goals*, *activities* and *outcomes.* Rehabilitation has been a long-term need of stroke patients and studies show stroke patients’ unmet needs regarding rehabilitation and activities of daily living [27, 53–55]. In the tool, patients are able to independently or together with a neurology team set their goals and activities based on their identified problems. The rehabilitation plan includes both simple and SMART (Specific, Measurable, Achievable, Realistic, Timely) goals as rehabilitation is a complex process and requires clear, specific and personal goals for a patient [56]. We chose to have both types of goals to provide patients opportunity to define their simple goals (e.g. being able to talk on phone) as well as their SMART goals (e.g. being able to walk 50 m within 10 min) in which they are able to measure and follow their progress using goal attainment scaling (GAS) [57]. To use the GAS method, the expected goal, its importance and difficulty level and expected outcomes should be defined. In addition, the baseline for the condition of the patient before the training and the GAS calculations should be done [57].

**Fig. 1:**
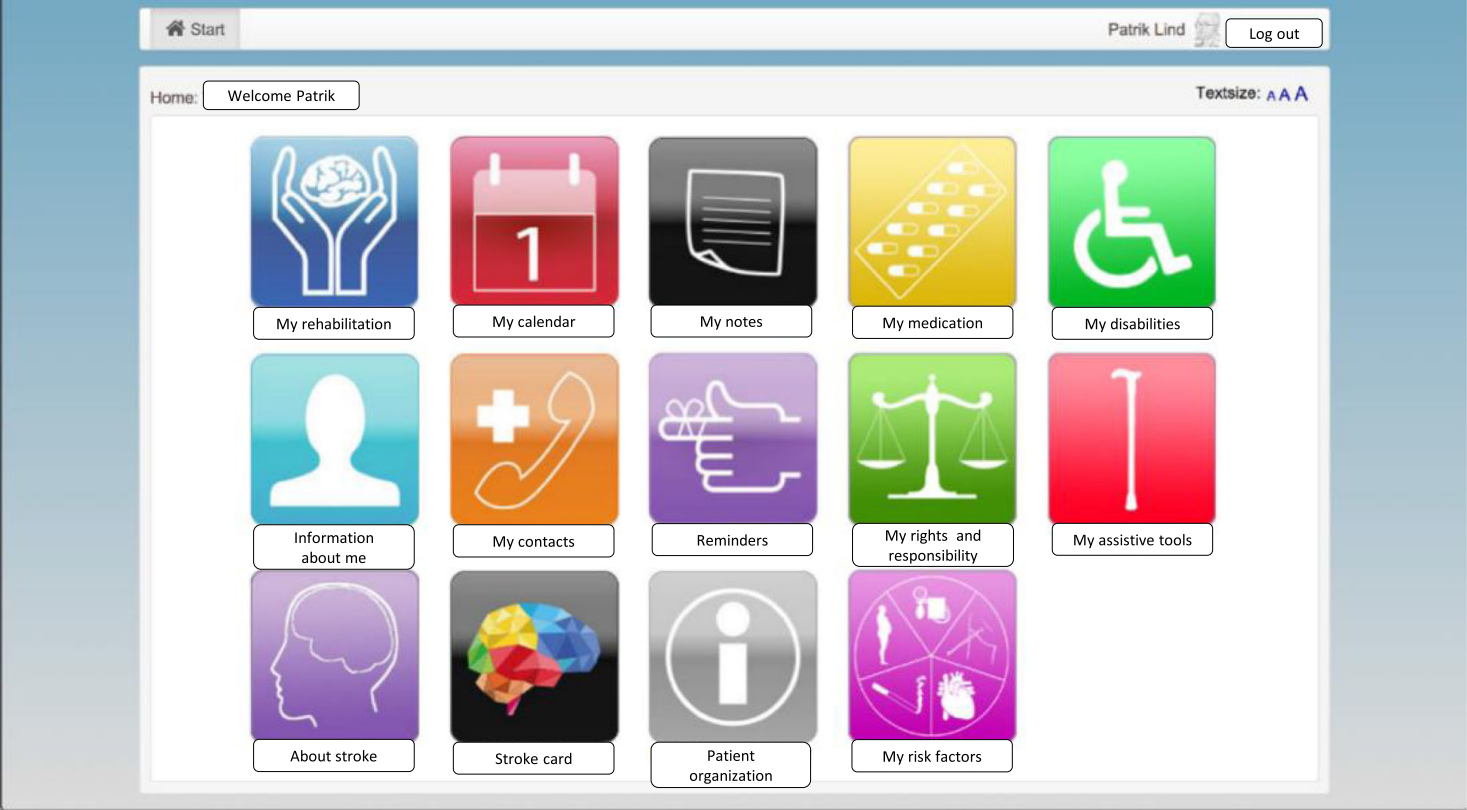
The web-based care and rehabilitation planning tool for post-discharge stroke patients. The name which is displayed in the figure is a fictitious person not an existing patient

The tool also includes some other features/eHealth services such as *calendar, patient’s notes, medication list*, *list of disabilities*, *and general information about the patient, contact information to care providers*, *reminder*, *patient’s rights and responsibilities, assistive tools*, *general information about stroke, patient associations, patient’s organisations and patient’s risk factors.* Figure 1 illustrates the eHealth services embedded in the electronic care and rehabilitation planning tool. The figure is a screenshot of the care and rehabilitation planning tool ‘My Care Plan’. The figure and the icons inside the figure have been developed in our previous studies [27–29].

Providing appropriate information to stroke patients has been the focus of several studies and the eHealth services in the care and rehabilitation planning tool are based on the patients’ information need explored in some of the previous studies [27, 58, 59]. The tool was introduced to patients and a neurology team in a primary care centre in Stockholm County Council. An evaluation of the tool based on the Unified Theory of Acceptance and Use of Technology (UTAUT) was done with the care professionals in the neurology team [60]. A key issue brought up by both patients and care professionals was that to be truly useful, the tool needs to be integrated with current HIS in use to reduce double documentation and manual transfer of information between systems. The HIP platform provides an opportunity to connect these types of eHealth services to current HIS, however since HIP was not available when this tool was designed the resources were not used. Since the tool was designed without having access to both opportunities and limitation of the HIP resources and the national HIE platform, this study aims to explore how this infrastructure meets the patients’ information needs as identified and designed for in the “My Care Plan” tool and thereby describe requirements that HIE platforms, such as the Swedish one, need to meet.

## Methods

**Fig. 2:**
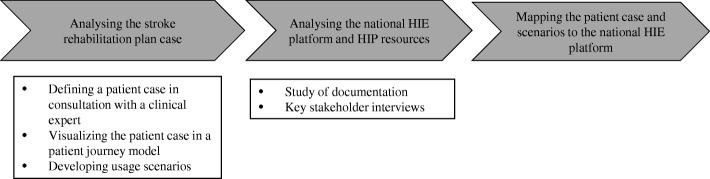
An overview of the process of collecting and analysing data in this study

A single case study approach [61, 62] was adopted for this study as an in-depth understanding of the subject was needed. With the aim of exploring opportunities and limitations of the national Swedish eHealth infrastructure, we used the concrete case of the post-discharge stroke care and rehabilitation planning tool and identified patients’ needs as the starting point for analysis. A stroke coordinator in Stockholm and a key stakeholder familiar with the architecture of the national HIE platform were participating in this study and provided us with valuable information. We studied the Swedish national HIE platform as it enables communication between health information systems and eHealth services such as ‘My Care Plan’ and different source systems currently in use in healthcare. To our knowledge, there is a lack of studies focusing on information flow and communication between different information systems using the Swedish national HIE platform. We took our starting point in our understanding of stroke patients’ needs before analysing documents and materials related to APIs (Application Programming Interfaces) currently available through the national HIE platform and relevant service contracts. Finally, we used a patient journey model to map the needs described in the scenarios to the resources available in the national HIE platform. Figure 2 illustrates an overview of the process of collecting and analysing data in this study.

### Analysing the stroke rehabilitation plan case

We chose to ground our analysis in a concrete patient case, describing a fictive stroke patient’s journey through healthcare, (we called her Anna, description in section 3.1.1.), where information is created and stored in different health information systems, and where the patient wishes to access information and eHealth services. We base this analysis on previous research as described below.

#### Defining a patient case in consultation with a clinical expert

In our previous study [27] several personas [63, 64] were created, based on data collection in focus group interviews with stroke patients. A persona is a tool used in software development projects to create understanding for the users as it is a concrete, yet fictive example of the user group. To gain a deeper understanding of how necessary health information for a patient is transferred in different situations, a fictive patient case with a pseudo-name was created based on the previous personas. To enrich the case, an unstructured interview [61] with a stroke coordinator in Stockholm County Council was also performed. The interview was audio recorded and lasted approximately one hour. Subsequently, a fictive patient record was prepared by the stroke coordinator and was used in the modelling process.

#### Visualizing the patient case in a patient journey model

**Fig. 3:**
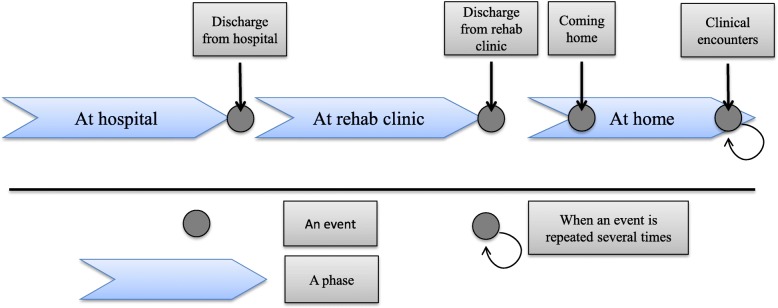
An overview of stroke patients’ journey model

Post-discharge stroke patients’ journey and their care and rehabilitation processes were modelled in our previous studies [27, 52]. Patient journey refers to “the experiences and processes the patient goes through during the course of a disease and its treatment” [65]. It provides an overview of how e.g. post-discharge stroke patients experience their care and rehabilitation processes. In this study, we used the general patient journey model as a basis to visualize our patient’s journey through post-discharge healthcare in more detail. The general patient journey model consists of several events and phases. Several events may be incorporated in a phase which is extended over time. Figure 3 is modified from the post-discharge stroke patients’ journey modelled in our previous study and is based on the patient’s case that will be described in the results section in this paper [27].

#### Developing usage scenarios

Different usage scenarios were created to represent our stroke patient in different situations with different problems and needs. The scenarios were used to illustrate different situations in which the patient needs to have access to different types of administrative and/or health related information. The first scenario provides an example of basic access to health related information through the national HIE platform. The second scenario describes an interactive access to more complex health related information and the third one focuses on the access to information which is currently not stored in a structured way in the EHR systems. The information flow in these scenarios was studied between some of the eHealth services proposed in the care and rehabilitation planning tool and different HISs such as EHRs and eHealth services connected to the national HIE platform. The scenarios are based on the stroke patients’ information needs identified in our previous study [27]. In this study, we defined scenarios in which patients are in need of having access to general but necessary information such as their medication list and assistive tools. In addition, we focused on scenarios where patients wish to be able to be actively involved in updating and sharing their health related information with the care professionals using e.g. their rehabilitation plan. We consciously choose to design the scenarios mentioned above to cover patients’ different needs of accessing and sharing information.

### Analysing the national HIE platform and HIP resources

The second part of the study involves understanding, describing and analysing the resources available through the national HIE platform and HIP.

#### Study of documentation

The materials were mainly obtained through Inera [26] and HIP websites [66]. Regulatory framework for the national HIE platform and relevant service contracts such as ‘GetMedicationHistory’ and ‘GetCarePlans’ have been studied from the website for legal framework for interoperability/information exchange in health care [67]. Availability of APIs and service contracts for different information needs were studied. Relevant APIs and Service contracts were selected and studied in details to get a deep understanding of the type of information that they contain. In order to get an overview of which health information can be accessible through the national HIE platform, the information studied in different APIs and service contracts were mapped with the post-discharge stroke patients’ information needs.

#### Key stakeholder interviews

To enrich the understanding obtained from the document analysis, a key stakeholder familiar with the architecture of the national HIE platform provided us with relevant information about different service contracts and APIs available through HIP. The interview lasted about one hour. The material was then used for analysing different scenarios regarding the information flow between different eHealth services in the care and rehabilitation planning tool with different HISs and eHealth services.

### Mapping the patient case and scenarios to the national HIE platform

Stroke patients’ information needs were identified in a previous study [27], and formed the basis for the design of “My Care Plan”. In this study, we compared the needs identified with the APIs and service contracts currently available through the national HIE platform to determine what information needs can and cannot be met by connecting to the national HIE platform today.

In addition, we used the 3 usage scenarios we designed to cover different types of information and interactions to visualize how these types of information can or cannot be accessed through the national HIE platform. The scenarios describe situations related to accessing, updating, and sharing health related information.

## Results

The results are divided into different sections: (1) a patient case based on the previous personas and a mapping of the journey model with the patients’ needs, (2) an overview of the available service contracts and APIs, (3) different usage scenarios with an analysis of the information flow between the care and rehabilitation planning tool and different source systems through the national HIE platform.

### Patient case and visualization of information flow in a patient journey

To be able to visualise the information flow for some of the potential eHealth services in our study we have used a patient case in which different information is needed. A short description of the patient case followed by the visualisation of information flow is presented in this section.

#### Fictive patient case

In this section we are describing our fictive patient that we will refer to as ‘Anna’. Anna is 46 years old. She works as a consultant and has two children (10 & 16 years old). She suffers a stroke after a workout at the gym with paralysis in the right side. She has mild nausea and starts slurring. Her husband makes a FAST (face, body, speech, time) test and calls SOS alarm. She had been in contact with health care professionals due to her previous health issues which are high blood pressure and high cholesterol level. Table 1 illustrate the information that has been documented in Anna’s record at hospital at the stroke occurrence, at the rehabilitation clinic, and at home by the care professionals in the neurology team as well as the communication requirements between different instances.

**Table 1:** An overview of the information documented in Anna’s electronic health record

At the hospital	At the rehab clinic (two months)	At home with the neurology team
- Admission cause: Mild nausea, paralysis of right side, slurs - Social: lives with husband and two children - Earlier disease: Hypertension and dyslipidaemia. Has contact with healthcare - Tobacco: not tested - Alcohol: not tested - Driving: has a driving license - Current drugs: Simvastain 20 mg, Enalapril 10 mg - Investigations: Computer tomography, bleeding omitted - Assessment: Reduced function in the right side, arm and ben, Aphasia, hard to understand. Patient is concerned about her situation and how it will affect her children. - Action: Blood thinning and clot-dissolving medicine. - Computed tomography, bleeding excluded. - Physiotherapist: The patient feels slightly sick for a jog and becoming paralyzed in the right side. The patient cannot walk without support. Gets up with easy-care support. - Occupational: MOCA test. Patient does not know what day it is and not where she is. - Speech therapist: Boston Naming Test - have great difficulty naming several of the pictures. The patient has a good understanding of the situation, but it is difficult to follow long complicated instructions. - The patient’s children have also talked to the counsellor. - Speech therapist, Physiotherapist, and occupational therapist contact - Continued counsellor contact - Doctors. The patient is discharged from the clinic. She has improved during the hospital stay. Referral to a rehabilitation clinic.	- Admission Cause: Weakness in the right arm and leg. Aphasia. Do not remember everything that happened in the Emergency Department. - Occupational therapy - Cognitive screening, arm and hand exercise - Speech therapy language training and literacy classes - Neuropsychologist - investigation. The patient gets tired quickly, can only read short moments.	- Physical therapy - The patient has constant pain on right side and gets hurt by the slightest movement. Despite this patient practices with the physiotherapist. She also exercises three hours / day. She is informed that even everyday tasks are training. - Occupational therapy - kitchen training, as well as shopping. - Speech Pathology - language training as well as reading and writing exercises. - Summary of neurology team’s efforts. The patient now has a diary, which she can enter the date of exercise and health visit. The patient gets still tired easily and must therefore rest frequently. - It is important for her to plan their activities so that she can manage her daily work. - The patient can now walk longer distances and can use her right hand. She has improved linguistically - may now take part in conversations with several people. She also reads and writes better, but not enough for her to start working. Fatigue also prevents her from long working days. - She is concerned about their hidden disabilities such as brain fatigue and personality change. - Patient is considering why she was diagnosed with stroke. - Neurology team provides continuous information about stroke and its consequences. The patient also receives explanation of what and why she should train and motivate her to continue training. Patient is also encouraged to re-connect with the family doctor. Neurology team is also in contact with family doctor during the treatment period. - Patient is also encouraged to contact the patient associations who are in the locality and informed of SMIL (Stroke in the middle of life). SMIL also has a Facebook group where you can talk with others who have had a stroke. The patient is also informed that she can through the family doctor apply for specialist planned rehabilitation.

After discharge from the hospital and the rehabilitation clinic, Anna receives care and rehabilitation from care professionals at her primary care centre and a neurology team. Anna wants to have a comprehensive picture of her rehabilitation activities, physician appointments and the neurology team’s home visits to be able to plan her day and track her progress. The neurology team has provided Anna with some information about the care and rehabilitation planning tool that she can use online at home. Therefore, Anna start using the tool to plan her personal rehabilitation activities, get access to the neurology team’s rehabilitation plan and also information about her medication and assistive tools.

#### Mapping of the journey model with the patient information needs

**Fig. 4:**
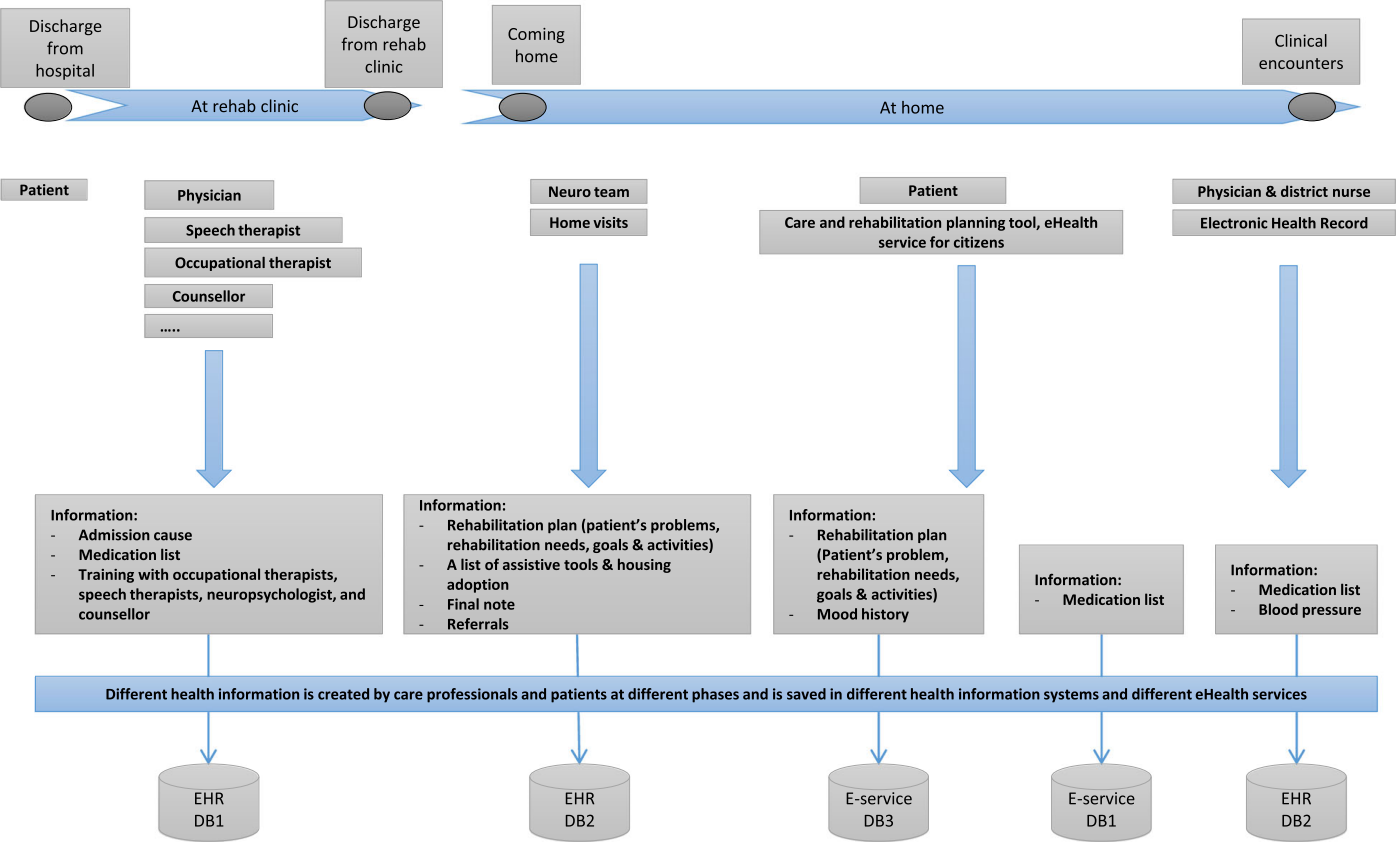
An overview of patient journey, with example on different health information which is created at different phases of the patient journey and is saved in different health information systems and eHealth service systems

To visualize when information is created in during the post-discharge journey, we have mapped the patient journey model to the patient case to indicate use of different tools and creation of information. In Fig. 4, we visualize Anna’s care and rehabilitation process to increase understanding of both actors involved and information systems in use. An overview of Anna’s journey, different care professionals involved in her care and rehabilitation processes, different health information systems used by different care professionals and necessary health information documented in several source systems are illustrated in Fig. 4.

According to the patient case described above, different information amount is created during different phases of Anna’s journey. Several care professionals are involved in creating and documenting this information which is stored in Anna’s EHRs. Use cases in the figure above illustrate some of the activities each actor e.g. care professionals in the neurology team, the patient, and physician and district nurse performs within the Anna’s care and rehabilitation journey. (See Additional file 1).

### Available service contracts and APIs

Necessary information and potential eHealth services were identified in our previous study [27] for design of the care and rehabilitation planning tool for discharge stroke patients and are presented in Table 2.

**Table 2:** A presentation of post-discharge stroke patients’ information needs related potential eHealth services (adopted from [27]), an overview of available service contracts, APIs based on the post discharge stroke patients’ and information needs and their need of eHealth services

Necessary information identified in previous studies	Potential eHealth services	Service contract	APIs available for citizen services and services for healthcare professionals	Comments
An overview of what has happened	My discharge notes	Not Available	Not available	
An overview of what is planned	My calendar	Available	Not available	
	My referrals	Available	Available (Referral status for citizen and Referral response for care professionals)	Referral status is available for both citizen services and care professionals
An overview of health related information	My health information (diagnosis, symptoms, disease history)	Available	Available (diagnosis)	Diagnosis is available both for citizen services and care professionals.
	My medication	Available	Available (Medication data for citizen, Medication list for care professionals)	Available for both citizen services and care professionals
An overview of risk factors and disabilities	My risk factors	Available	Available	Since the risk factors are documented in patient’s EHR, the information is possible to retrieve through ‘GetCareDocumentation’ Available for both citizen services and care professionals
	My disabilities	Available	Available (PADL + Disability for citizen, Function status for care professionals)	
An overview of care providers contact information, their specialties, and responsibilities	My care contacts	Available	Available (contact information for healthcare)	Available for both citizen services and care professionals
Measurement and documentation of health related parameters	My diary	Not available, patient’s own information	Not available	
Support for sharing of personal observations				
An overview of the goals and planned activities	My rehabilitation plan	Available	Available (My care plan)	My care plan also include rehabilitation plan. However, it is only available for care professionals
Rights and responsibilities regarding e.g. continued rehabilitation, assistive devices and health insurance	My rights & My responsibilities	Not available, available publically	Not available	
An overview of patients associations and social networks	My patient organizations and support associations	Not available, Links to 1177 or insurance agency	Not available	
	My assistive tools	Not available	Not available	
	Information about me e.g. my personal data, my contact details	Available	Not available	
	About stroke	Not available, common information	Not available	
	Reminders	Not available, Patient’s own information	Not available	

To be able to explore the opportunities and limitation of accessing and interacting with some health information through the Swedish national HIE platform the availability of APIs have been studied. In addition, the availability of service contracts that makes the integration possible for our identified potential eHealth services has been studied and presented in Table 2. All necessary information addressed in our care and rehabilitation tool ‘My Care Plan’ is not retrievable from different health information systems as service contracts and relevant APIs for all eHealth services are not available. This will result in limited interoperability between My Care Plan and other health information systems and lack of patient participation in the care and rehabilitation processes.

The service contract ‘GetCarePlans’ includes ‘typeOfCarePlan’ in which different types of care plans such as Coordinated individual plan, Coordinated plan at discharge, Care plan, *Habilitation plan*, *Rehabilitation plan*, *Standardised care plan* are included. In addition, service contracts “GetCareContacts” and “GetSubjectOFCareSchedule” include information about patient’s appointments with healthcare professionals and calendar.

The participants in our previous studies wished to have access to their discharge notes ‘My discharge notes’ from hospital. Discharge notes are documented in the patient’s health record and the service contract “GetCareDocumentation” is available for it. Information about medication list and disabilities is accessible through the potential eHealth services ‘My medication’ and ‘My disabilities’ as it is documented in the patient’s EHR and there are service contracts ‘GetMedicationHistory’ and ‘GetFunctionalStatus’ available.

Certain information that the patients wanted access to, such as ‘Information about me’, ‘My diary’ and ‘Reminders’, are specific information about the patient and his/her planned activities that are not documented in patient’s health record but can rather be created by the patients themselves. In addition, there is no service contract developed for ‘My rights and responsibilities’, ‘About stroke’, ‘My assistive tools’, and ‘Patient organisations’ as they are general information and are available publicly through the webpage 1177 vårdguiden, a national Swedish website and telephone service which provides information, counselling and services in healthcare [34].

Patients’ desired information about contact information for different healthcare providers is also available using eHealth service ‘My care contacts’ as a service contract called ‘MyCareUnits’ has been developed.

Patient also wished to have access to information about their risk factors ‘My risk factors’ through the care and rehabilitation planning tool. Since the information is documented in the EHR, the information is possible to retrieve from ‘GetCareDocumentation’. However, since the information is likely not defined or structured as stroke risk factors in the EHR, it will be up to the application accessing the information to specify what factors are considered risk factors for stroke and identify them in the information delivered through ‘GetCareDocumentation’. As risk factors differ for different conditions, a general risk factor service contract is likely not feasible. General information about stroke risk factors is public and accessible through 1177 Vårdguiden.

### Usage scenarios and analysis of the information flow

We have identified three different scenarios that represent different types of information access; (1) basic access to health related information, (2) interactive access to complex health related information, and (3) access to borderline health related information. Each scenario is described in further detail below, and the information flow is visualised in this section. Each scenario is based on the patient case described above, and activities that the patient may want to perform to access and interact with information based on the needs analysis.

#### Scenario I: Anna wants to have access to the medication list documented in her health record system

This scenario provides an example of basic access to health related information through the national HIE platform.

Anna logs in to the care and rehabilitation planning tool and chooses the ‘My medications’ eHealth service to check the given dosage on her blood-thinning drug. To retrieve the medication list from the source systems, a request is sent from the eHealth service ‘My medications’ (the service consumer) in the care and rehabilitation planning tool to the national HIE platform. The visualisation platform checks the authentication, access control, routing and other validation for sending the message further to the service provider. It also returns the reply message whether it is a normal response or an error message to the service consumer.

The aggregation service is a component in the national HIE platform and it provides the service consumer with a compiled response by contacting a number of service providers, based on information in the engagement index. The aggregation service compiles the responses that are received from individual service providers and creates an aggregate response.

**Fig. 5:**
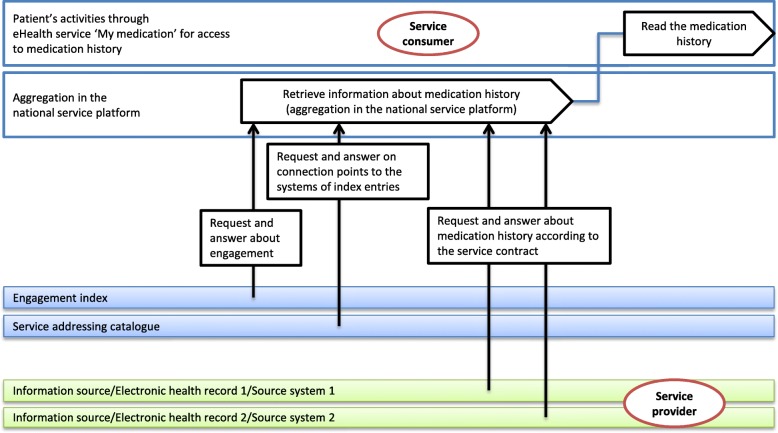
Addressing the call to aggregation service from a service consumer e.g. ‘My medication’. The integration between a service consumer and a service provider through the national HIE platform. Information source/EHRs/source systems 1&2 provides health related information to the national HIE platform according to predefined service contracts and the service consumer in this case ‘My medication’ requests information from the service providers through the platform

The engagement index assists the aggregation platform by registering the service producers that have data of a specific type for a particular person. Through the service contract “GetMedicationHistory”, the medication history is available for the service consumer. A response message is sent through the visualisation platform to the service consumer, and Anna is able to read her medication history (Fig. 5).

Since Anna has had medications prescribed both at the regional hospital, and at her local primary care centre which uses a different EHR system, the engagement index will indicate that medication information is available from two different service providers. Of course, for Anna to have access to all her prescribed medications, both care providers must have connected their EHR systems as service providers to this particular service contract. If one has not, Anna will only have access to parts of her prescribed medications. In Sweden, there are 21 county council, 290 municipalities, and a growing number of private healthcare providers. Currently, all 21 county councils are connected to the HIE. Fewer of the municipalities and private care providers are connected today but the number is increasing and there are currently more than 70 million producer calls made monthly [38]. However, the decision which information to disclose to the patient and when is made at a regional/county council level. As a result, information access may vary greatly depending on the region the patient was treated in.

#### Scenario II: Anna wishes to have access to the rehabilitation plan using the eHealth service ‘my rehabilitation’, update and share it with the care professionals in the neurology team

This scenario describes an interactive access to more complex health related information.

**Fig. 6:**
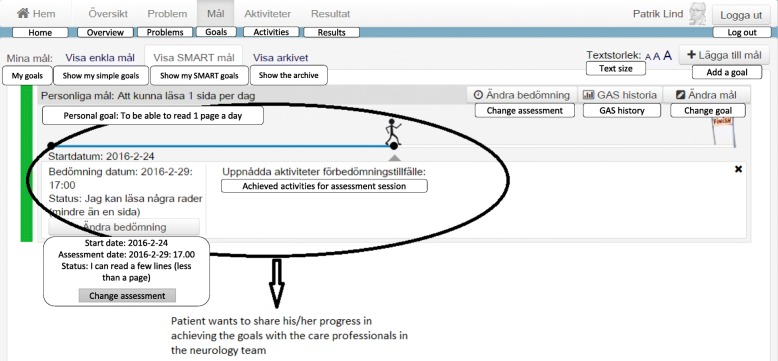
Updating and sharing the rehabilitation plan with the care professionals in the neurology team

The procedure for accessing information through the national HIE platform is the same for the rehabilitation plan as for the medication list. Anna is able to access the rehabilitation plan documented in her EHR as the related service contract ‘GetCarePlans’ is available. Although information can be accessible from the source systems through the national HIE platform, few EHR systems are also connected as service consumers. This means that if new rehabilitation plan data is created in ‘My rehabilitation plan’ the healthcare professionals EHR system will likely not be prepared to access this information as a service consumer. There is therefore limited possibility for Anna to update the rehabilitation plan and share the new version with the care professionals in the neurology team. When Anna has performed some planned activities she can report this in the ‘My rehabilitation plan’ eHealth service, but the information about her performed activities and partial goal achievement will not be accessible for her neurology team through their EHR. Therefore, there is currently no support for her to send the result of e.g. her goal assessment to the neurology team (Fig. 6).

In order for Anna to be able to share her updates with her neurology team, their system would need to be adapted to be able to receive such information through the national HIE platform, and a new service contract for reporting activities would possibly need to be implemented, where the eHealth service ‘My rehabilitation plan’ acts as service provider and the neurology teams EHR acts as service consumer.

#### Scenario III: access to the list of assistive tools using eHealth service ‘My assistive tools’

Finally, in the third scenario we explore access to information which is currently not stored in a structured way in the EHR systems.

Anna has been prescribed a number of assistive tools to help manage living at home after the stroke. By logging in to the eHealth service ‘My assistive tools’ she would like to see a list of the tools such as her bath chair, anti-slip rubber mat and sock aid that she has been prescribed, and information about other tools that are available. It is however not possible for Anna to retrieve information about her assistive tools from her health record through the national HIE platform as there is no service contract developed for accessing the assistive tools. Whenever an assistive tool is prescribed by a healthcare professional, this should be documented in the EHR. However, this is not done in the structured fashion of medication prescriptions, but is rather stored in free text in a medical record note. This is very likely one of the reasons why no service contract has been implemented for this information; it is difficult to find the information in the record when it is not structured.

More structured information about the assistive tools that Anna has been prescribed could be available in other health information systems, e.g. in the prescription database at the County Council’s centre for assistive tools management, or in social care records at the municipality, who are e.g. responsible for making assessments of changes required in the home environment. However, these information sources are not yet connected as service providers to the national HIE platform.

## Discussion

Accessing personal health information from multiple source systems has not yet been widely adopted. However, a number of studies have focused on giving patients access to their EHRs [5–7, 68], studying the care professionals experiences [69] and investigating the effects of providing online access as well as its barriers and facilitators for providing patients online access to their health information [4].

The results of these studies show that despite the limitations in accessing health information online and some objections and concerns by care professionals, patients and care professionals are positive towards this reform. This is in line with our previous studies with both stroke patients and healthcare professionals who are overall positive towards patients getting access to their stroke related information online.

In this study we wanted to explore the opportunities of meeting the identified patient needs in the current Swedish health context. We therefore used the case of the care and rehabilitation planning tool to explore the opportunities and limitations of accessing and interacting with necessary health information through the Swedish national HIE platform. However, the results of this study can be used in other countries that are about to establish a similar platform for the exchange of health information. There are certainly patients in other parts of the world who want to have access to their health information and also be able to share their information with their health care professionals. Our use cases are not restricted to the Swedish or Scandinavian context. The experience gained in this study may, therefore, be useful in other contexts outside Sweden.

The results show that despite the fact that the national HIE platform provides opportunities for patients to access necessary health information from different source systems using eHealth services, there is still no support for patients sharing information with their care professionals. This would require redesign of current health information systems, e.g. EHRs, to enable interactive health information exchange between patients and healthcare professionals. In addition, the results show that there is necessary information which is not retrievable as it is either stored in electronic health records and eHealth services which are not connected to the Swedish national HIE platform or there is no service. Healthcare professionals today often work in a stressful environment, and express that they cannot take the time to use yet another health information system in their daily work [60]. It is therefore imperative to ensure that eHealth services that are designed for patients are able to communicate with the HIS used by healthcare professionals. The national HIE platform provides the building blocks for allowing such interoperability, but due to the lack of EHR systems currently acting as service consumers we have yet to achieve the type of interoperability described in scenario II. Therefore patients can still not be actively involved in their care and rehabilitation processes using eHealth services to share their health information with different care professionals.

Although healthcare is moving towards a patient-centred care in which patient participation and patient empowerment are the key concepts, there is still very little support for patients to provide input into their EHRs. Currently, only the care professionals have the opportunity to document, update, and share patient’s health information.

Results in this study show that while there are APIs and service contracts available for a lot of necessary health information, patients have broader information needs in which no service contract neither APIs have been developed. Therefore, there is a need of developing more service contracts based on the patients’ information needs. In addition, there is a need of connecting more source systems to the national HIE platform as a lot of patient’s necessary information is documented in other health information systems than patient’s EHR. More importantly, the national vision of EHR systems and other major sources for health information to also act as service consumers, accessing patient-created data, through the national HIE platform needs to be achieved.

Exchanging health information from a personal health record (PHR) into an electronic health record (EHR) and vice versa, is described in Integrating the Health Enterprise (IHE) technical framework in (XPHR) integration profile [17]. However, future studies are needed to investigate the opportunities and limitation in information exchange between eHealth services and different health information systems where patients wish to share information with the care professionals in Sweden. Although the exchange of a huge wealth of health information is possible between different health information systems through international standards such as the HL7 suite of standards, there is still limited support for accessing all patient’s information needs identified and discussed in this and previous study [27].

This paper presents a theoretical analysis of opportunities to retrieve necessary information through the national HIE platform, but there is still limited knowledge about how information will actually appear and be presented in different eHealth services used by patients. The availability of service contracts and API’s does not guarantee that information is documented as expected in the source systems, and this potential lack of data quality can have a detrimental impact on the useful of eHealth services designed to access and display it. Therefore, to be able to design appropriate eHealth services, future studies are needed to focus on information structure, and actual information stored, in different source systems and eHealth services. However, designing and developing appropriate eHealth services and health information systems is not only about information structure and presentation of information to healthcare providers, patients, and citizens. Providing useful eHealth solutions requires even opportunities of having a two way communication between different eHealth solutions. Therefore, it is of great importance to identify the information needs from both healthcare providers and patients and to develop appropriate service contracts and APIs for maintaining interoperability.

## Conclusion

The national Swedish HIE platform provides the building blocks needed to allow patient online access to their health information in a fragmented and distributed health system. However, the more complex interaction scenarios, where patients can provide information to their healthcare professionals using their professional tools, i.e. EHRs, or even update their health related information is not yet supported. In order to provide truly patient-centred and empowering eHealth solutions, is not only a technical problem, but a socio-technical. It will require collaboration between national agencies providing the infrastructure, health information system vendors adapting to new requirements of interactivity and interoperability, and perhaps even more important healthcare organizations and professionals adapting their way of working to include patient-created data in their work processes. Last but not least, patients need to be involved throughout the design and evaluation of eHealth services on both national and local levels to ensure that their needs for interoperability and information exchange are met.

## Additional file

Additional file 1: Use case diagram (neuro team, home visits). The diagram illustrates the interaction between different actors in a neuro team (counsellor, occupational therapist, speech therapist, and physiotherapist) with the electronic health record. Use case diagram (patient, care and rehabilitation planning tool, eHealth services for citizens). The diagram illustrates the interaction between a patient and the care and rehabilitation planning tool. Use case diagram (physician & district nurse, electronic health record). The diagram illustrates the interaction between a physician and a district nurse with the electronic health record. (DOCX 451 kb)

## Abbreviations

API: Application programming interfaces
CDA: Clinical document architecture
DICOM: Digital imaging and communications in medicine
EHR: Electronic health record
FAST: Face, body, speech, time
FHIR: Fast health interoperability resources
GAS: Goal attainment scaling
GEHR: Good european health record
HIE: Health information exchange
HIP: Health innovation platform
HIS: Health information system
HL7: Health Level 7
ICT: Information and communication technology
IHE: Integrating the healthcare enterprise
ISO/EN: International standard
PHR: Personal health record
RIM: Reference information model
SMART: Specific, measurable, achievable, realistic, timely
SMART: Substitutable medical applications and reusable technologies
UTAUT: Unified theory of acceptance and use of technology
XPHR: Exchange of personal health record
