# Supplementary material for: Accessing and sharing health information for post-discharge stroke care through a national health information exchange platform - a case study
Source: BMC Med Inform Decis Mak. 2019 May 3;19:95. doi: 10.1186/s12911-019-0816-x (PMC6500022; doi:10.1186/s12911-019-0816-x)
Supplement: Supplementary file 1 — Use case diagram (neuro team, home visits). The diagram illustrates the interaction between different actors in a neuro team (counsellor, occupational therapist, speech therapist, and physiotherapist) with the electronic health record. Use case diagram (patient, care and rehabilitation planning tool, eHealth services for citizens). The diagram illustrates the interaction between a patient and the care and rehabilitation planning tool. Use case diagram (physician & district nurse, electronic health record). The diagram illustrates the interaction between a physician and a district nurse with the electronic health record. (DOCX 451 kb) [file 12911_2019_816_MOESM1_ESM.docx]

# Additional file 1

**Neuro team**

**Home visits**


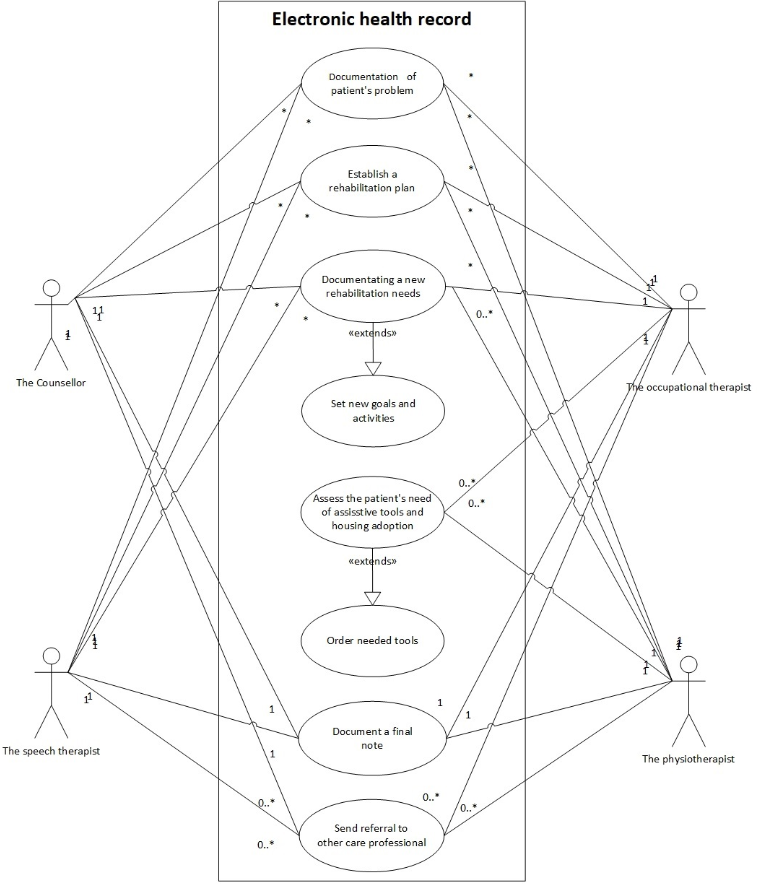


**Care and rehabilitation planning tool, eHealth service for citizens**

**Patient**

**Physician & district nurse**

**Electronic Health Record**
